# Supplementary material for: High-throughput analysis of single human cells reveals the complex nature of DNA replication timing control
Source: Nat Commun. 2022 May 3;13:2402. doi: 10.1038/s41467-022-30212-y (PMC9065153; doi:10.1038/s41467-022-30212-y)
Supplement: Supplementary file 3 — Reporting Summary [file 41467_2022_30212_MOESM3_ESM.pdf]

nature portfolio

Corresponding author(s): Amnon Koren

Last updated by author(s): Apr 12, 2022

Reporting Summary

Nature Portfolio wishes to improve the reproducibility of the work that we publish. This form provides structure for consistency and transparency in reporting. For further information on Nature Portfolio policies, see our Editorial Policies and the Editorial Policy Checklist.

Statistics

For all statistical analyses, confirm that the following items are present in the figure legend, table legend, main text, or Methods section.

n/a	Confirmed	
☐	☒	The exact sample size (*n*) for each experimental group/condition, given as a discrete number and unit of measurement
☐	☒	A statement on whether measurements were taken from distinct samples or whether the same sample was measured repeatedly
☒	☐	The statistical test(s) used AND whether they are one- or two-sided<br>*Only common tests should be described solely by name; describe more complex techniques in the Methods section.*
☒	☐	A description of all covariates tested
☒	☐	A description of any assumptions or corrections, such as tests of normality and adjustment for multiple comparisons
☒	☐	A full description of the statistical parameters including central tendency (e.g. means) or other basic estimates (e.g. regression coefficient) AND variation (e.g. standard deviation) or associated estimates of uncertainty (e.g. confidence intervals)
☒	☐	For null hypothesis testing, the test statistic (e.g. *F*, *t*, *r*) with confidence intervals, effect sizes, degrees of freedom and *P* value noted<br>*Give P values as exact values whenever suitable.*
☒	☐	For Bayesian analysis, information on the choice of priors and Markov chain Monte Carlo settings
☒	☐	For hierarchical and complex designs, identification of the appropriate level for tests and full reporting of outcomes
☒	☐	Estimates of effect sizes (e.g. Cohen's *d*, Pearson's *r*), indicating how they were calculated

Our web collection on statistics for biologists contains articles on many of the points above.

Software and code

Policy information about availability of computer code

Data collection	No software was used for data collection.
Data analysis	Data analysis was performed using custom python3 (v3.6.8), MATLAB (v2019a) and awk (v4.0.2) scripts. Open source components used within the pipeline were: seqtk (v1.2-r102-dirty), bwa (v0.7.17-r1188), PicardTools (v2.9.0), and samtools (v1.11). The following python libraries were used: numpy (v1.19.0), pandas (v1.0.5), pysam (v0.16.0.1), h5py (v2.10.0), argparse (v1.1), ctypes (v1.1.0), Bio (v1.77). All scripts are available at https://github.com/TheKorenLab/Single-cell-replication-timing.

For manuscripts utilizing custom algorithms or software that are central to the research but not yet described in published literature, software must be made available to editors and reviewers. We strongly encourage code deposition in a community repository (e.g. GitHub). See the Nature Portfolio guidelines for submitting code & software for further information.

Data

Policy information about availability of data

All manuscripts must include a data availability statement. This statement should provide the following information, where applicable:

- Accession codes, unique identifiers, or web links for publicly available datasets
- A description of any restrictions on data availability
- For clinical datasets or third party data, please ensure that the statement adheres to our policy

Sequencing data generated in this manuscript were deposited at the Sequence Read Archive under accessions PRJNA770772 [https://www.ncbi.nlm.nih.gov/sra/?term=PRJNA770772] (single-cell) and PRJNA419407 [https://www.ncbi.nlm.nih.gov/sra/?term=PRJNA419407] (bulk). Bulk-sequencing replication timing profiles used for comparison are available at http://www.thekorenlab.org/data. The human reference genome assembly humanG1Kv37 used in this study was downloaded

from the International Genome Sample Resource [http://ftp.1000genomes.ebi.ac.uk/vol1/ftp/technical/reference/human_g1k_v37.fasta.gz]. Source data from the FACS sorting of GM12878 is provided with the manuscript.

Field-specific reporting

Please select the one below that is the best fit for your research. If you are not sure, read the appropriate sections before making your selection.

☒ Life sciences ☐ Behavioural & social sciences ☐ Ecological, evolutionary & environmental sciences

For a reference copy of the document with all sections, see nature.com/documents/nr-reporting-summary-flat.pdf

Life sciences study design

All studies must disclose on these points even when the disclosure is negative.

Sample size	For each library, we input ~5000 cells, with the expectation that we would yield ~800 cells after library preparation (~1/6 yield, per 10x Genomics) and that ~20% of the captured cells in an asynchronous library would be in S phase. Thus, we expected to recover ~150-200 replicating cells. This number was selected to achieve at least 50 reads per Mb in each cell at the desired library sequencing coverage, and in order to recover multiple cells per percentile of S phase.
Data exclusions	Computationally-identified "single cells" were excluded from analysis when read counts could not be assigned to two copy number states with a two-fold difference between them. This exclusion criterion was pre-established.
Replication	Experimental findings were reproducible across 8 GM12878 libraries and across the 10 cell lines under study.
Randomization	Randomization was not relevant to this study because there were no experimental treatments/conditions.
Blinding	Blinding was not relevant to this study because there were no experimental treatments/conditions.

Reporting for specific materials, systems and methods

We require information from authors about some types of materials, experimental systems and methods used in many studies. Here, indicate whether each material, system or method listed is relevant to your study. If you are not sure if a list item applies to your research, read the appropriate section before selecting a response.

Materials & experimental systems

n/a	Involved in the study	
☒	☐	Antibodies
☐	☒	Eukaryotic cell lines
☒	☐	Palaeontology and archaeology
☒	☐	Animals and other organisms
☒	☐	Human research participants
☒	☐	Clinical data
☒	☐	Dual use research of concern

Methods

n/a	Involved in the study	
☒	☐	ChIP-seq
☐	☒	Flow cytometry
☒	☐	MRI-based neuroimaging

Eukaryotic cell lines

Policy information about cell lines

Cell line source(s)	Lymphoblastoid cell lines (GM12878, GM12891, and GM12892) were obtained from the Coriell Institute for Medical Research. Embryonic stem cell lines (H1, H7, and H9) were obtained from the WiCell Research Institute (Madison, WI, USA). Tumor-derived cell lines (MCF-7, RKO, and HCT-116) were obtained from the American Type Culture Collection.
Authentication	All cell lines have been authenticated by whole-genome sequencing and SNP genotyping.
Mycoplasma contamination	All cell lines tested negative for mycoplasma.
Commonly misidentified lines (See ICLAC register)	None of the cell lines in this study are commonly-misidentified.

Flow Cytometry

Plots

Confirm that:

☒ The axis labels state the marker and fluorochrome used (e.g. CD4-FITC).

☒ The axis scales are clearly visible. Include numbers along axes only for bottom left plot of group (a 'group' is an analysis of identical markers).

☒ All plots are contour plots with outliers or pseudocolor plots.

☒ A numerical value for number of cells or percentage (with statistics) is provided.

Methodology

Sample preparation	Live cells were stained with Vybrant™ DyeCycle™ Green Stain (ThermoFisher Scientific, Waltham, MA, USA).
Instrument	BD FACSMelody™ Cell Sorter (BD Biosciences, Franklin Lakes, NJ, USA)
Software	BD FACSChorus™
Cell population abundance	The abundance and purity of post-sort populations was assessed using the in silico sorting method described in the manuscript (see, in particular, Figure 1 and Supplementary Figure 1).
Gating strategy	Cells were gated by DNA content into five fractions: G1, G2, early S, late S, and full S.

☒ Tick this box to confirm that a figure exemplifying the gating strategy is provided in the Supplementary Information.
